# Identify the potential target of efferocytosis in knee osteoarthritis synovial tissue: a bioinformatics and machine learning-based study

**DOI:** 10.3389/fimmu.2025.1550794

**Published:** 2025-02-27

**Authors:** Shangbo Niu, Mengmeng Li, Jinling Wang, Peirui Zhong, Xing Wen, Fujin Huang, Linwei Yin, Yang Liao, Jun Zhou

**Affiliations:** ^1^ Rehabilitation Medicine Center Hengyang Medical School, The First Affiliated Hospital, University of South China, Hengyang, Hunan, China; ^2^ Department of Rehabilitation, The First Affiliated Hospital, Hengyang Medical School, University of South China, Hengyang, Hunan, China; ^3^ Rehabilitation Laboratory, The First Affiliated Hospital, Hengyang Medical School, University of South China, Hengyang, Hunan, China

**Keywords:** knee osteoarthritis, efferocytosis-related genes, bioinformatics analysis, machine learning, immune infiltration

## Abstract

**Introduction:**

Knee osteoarthritis (KOA) is a degenerative joint disease characterized by the progressive deterioration of cartilage and synovial inflammation. A critical mechanism in the pathogenesis of KOA is impaired efferocytosis in synovial tissue. The present study aimed to identify and validate key efferocytosis-related genes (EFRGs) in KOA synovial tissue by using comprehensive bioinformatics and machine learning approaches.

**Methods:**

We integrated three datasets (GSE55235, GSE55457, and GSE12021) from the Gene Expression Omnibus database to screen differentially expressed genes (DEGs) associated with efferocytosis and performed weighted gene co-expression network analysis. Subsequently, we utilized univariate logistic regression analysis, least absolute shrinkage and selection operator regression, support vector machine, and random forest algorithms to further refine these genes. The results were then inputted into multivariate logistic regression analysis to construct a diagnostic nomogram. Public datasets and quantitative real-time PCR experiments were employed for validation. Additionally, immune infiltration analysis was conducted with CIBERSORT using the combined datasets.

**Results:**

Analysis of the intersection between DEGs and EFRGs identified 12 KOA-related efferocytosis DEGs. Further refinement through machine learning algorithms and multivariate logistic regression revealed UCP2, CX3CR1, and CEBPB as hub genes. Immune infiltration analysis demonstrated significant correlations between immune cell components and the expression levels of these hub genes. Validation using independent datasets and experimental approaches confirmed the robustness of these findings.

**Conclusions:**

This study successfully identified three hub genes (UCP2, CX3CR1, and CEBPB) with significant expression alterations in KOA, demonstrating high diagnostic potential and close associations with impaired efferocytosis. These targets may modulate synovial efferocytosis-related immune processes, offering novel therapeutic avenues for KOA intervention.

## 1 Introduction

Knee osteoarthritis (KOA) is one of the most prevalent degenerative joint diseases worldwide and affects approximately 530 million people globally (1). With an increase in the world’s aging population, the prevalence of KOA and its associated healthcare costs are steadily increasing, thereby substantially affecting the quality of life of patients with KOA (2). Despite advances in the understanding of the pathophysiology of KOA, current diagnostic and therapeutic strategies remain limited and primarily focus on symptom management rather than on addressing the underlying pathogenic mechanisms (3). Traditionally, KOA has been regarded as a degenerative disease predominantly driven by mechanical stress and characterized by cartilage degradation and loss. However, recent studies have revealed the involvement of a critical inflammatory component in nearly all joint tissues and have implicated complex mechanisms, such as immune responses, apoptosis, pyroptosis, and metabolic reprogramming, in KOA development (4, 5). Synovial inflammation plays a pivotal role in disease progression, and immune cell dysfunction in the synovium is recognized as a key mechanism that drives and sustains synovitis, resulting in persistent cartilage damage and degeneration (6). Understanding the functional abnormalities of immune cells in the synovium is crucial for elucidating the pathogenesis of KOA.

Efferocytosis refers to the process by which macrophages, other immune cells, and non-professional phagocytes, such as epithelial and endothelial cells, efficiently engulf and clear apoptotic cells (7). This process mediates alleviation of inflammation and restoration of tissue homeostasis by reducing the expression of proinflammatory cytokines and increasing the expression of anti-inflammatory cytokines such as interleukin (IL)-10 and transforming growth factor-beta (TGF-β). Efferocytosis plays a pivotal role in modulating inflammation and maintaining immune homeostasis, thereby suppressing inflammatory responses and promoting tissue repair (8, 9). In the synovial tissue of the knee joint, efferocytosis is mainly performed by synovial macrophages, which are key immune cells that maintain tissue homeostasis and prevent inflammation by clearing apoptotic cells and microdebris (10). Recent studies have demonstrated that the efferocytic function of synovial macrophages is considerably impaired in KOA patients (11). This dysfunction results in the inadequate clearance of apoptotic cells, leading to the activation of a prolonged immune response, chronic synovitis, and progressive joint degeneration (12). The depletion of synovial macrophages is also closely associated with pain symptoms in KOA (13). Although there is currently improved understanding of efferocytosis dysfunction in KOA, the specific mechanisms of efferocytosis in synovial tissues and their relationship with KOA-associated inflammation remain underexplored.

The precise molecular mechanisms linking efferocytosis to synovitis in KOA are unclear. This highlights the need to further investigate potential biomarkers and therapeutic targets that could facilitate the early diagnosis and intervention of KOA, eventually improving patient outcomes and reducing the societal impact of this common disease. Hence, the present study aimed to systematically identify key genes related to efferocytosis and analyze their relationship with different types of immune cells in KOA synovial tissues by integrating multiple publicly available datasets and applying approaches such as bioinformatics, machine learning methods, and CIBERSORT algorithm. Finally, three genes were identified as hub genes and confirmed by experiments. A preliminary diagnostic model was established and experimentally validated using animal models. This study may shed light on the relationship between efferocytic dysfunction and OA inflammatory mechanisms, thereby offering new potential avenues for future OA diagnosis and targeted therapies.

## 2 Methods

### 2.1 Data acquisition and preprocessing

Gene expression profiles of KOA patients and control subjects were obtained from the Gene Expression Omnibus (GEO) database (http://www.ncbi.nih.gov/geo). The GSE55235 (14), GSE55457, and GSE12021 (15) datasets from the GPL96 platform were used as the training datasets, and the GSE36700 (16), GSE82107 (17), and GSE77298 (18) datasets from the GPL570 platform were used as the validation datasets (**Table 1**). These datasets include synovial tissue samples from KOA patients and control subjects. The extracted data were normalized by log2 transformation. The microarray data were normalized using the normalize.quantiles function of the preprocessCore package in R software (version 3.4.1). The raw data were downloaded as MINiML files. The probes were converted to gene symbols according to the annotation information of the normalized data in the platform. Probes that matched multiple genes were removed from the datasets. The average expression value of the genes measured by multiple probes was calculated as the final expression value. For the data obtained from the same dataset and platform but in different batches, the removeBatchEffect function of the limma package in R software was used to remove batch effects. For the data obtained from different datasets or the same dataset but on different platforms, multiple datasets with common gene symbols were extracted, and different datasets or platforms were marked as different batches. The data preprocessing results were assessed using a boxplot. A principal component analysis (PCA) plot was generated to display the samples before and after batch effect removal (**Supplementary Figure 1**).

**Table 1:** Details of the datasets.

Type	Platform	Title	OA Samples	Control Samples
Training datasets	GPL96	GSE55235	10	10
		GSE55457	10	10
		GSE12021	10	9
Validation datasets	GPL570	GSE36700	5	0
		GSE82107	10	7
		GSE77298	0	7

#### 2.1.1 Identification of differentially expressed genes

DEGs between the KOA and control groups were identified using the limma package in R software (19). To minimize the risk of overlooking candidate genes and ensure significant differential changes, we set the following criteria: adjusted P-value < 0.05 and |log2 fold change| > 0.7. Volcano plots and heatmaps were generated using “ggplot2” and “pheatmap” packages, respectively, in R software to visualize the DEGs, which provided a comprehensive view of the gene expression landscape in KOA.

#### 2.1.2 Enrichment and correlation analysis

Gene Ontology (GO) analysis and Kyoto Encyclopedia of Genes and Genomes (KEGG) pathway enrichment analysis were conducted using the “clusterProfiler” package in R software to elucidate the biological functions and pathways significantly associated with the DEGs. All the abovementioned procedures were performed using the online tool available at: https://www.aclbi.com/. Protein-protein interaction (PPI) networks were constructed using the STRING database and visualized with Cytoscape to determine the interactions and correlations among the DEGs (20, 21).

### 2.2 Identification of efferocytosis-related DEGs

First, the efferocytosis-related genes (EFRGs) were identified from the GeneCards website (https://www.genecards.org/), a comprehensive database containing information on known genes across various species. Next, by using “efferocytosis” as the keyword, the relevant genes were retrieved from the KEGG database, a general-purpose bioinformatics resource containing molecular pathways and genomic information. To expand the selection of EFRGs, the gene lists from both databases were combined, and a Venn diagram was generated using the “VennDiagram” (version 1.7.3) package. From the DEGs identified in the GEO dataset, those related to efferocytosis were filtered out and designated as EFRDEGs. EFRDEGs were visualized using the “ggplot2” (version 3.4.4) and “ComplexHeatmap” (version 2.13.1) packages in R software.

### 2.3 Weighted gene co-expression network analysis

By using the WGCNA package (version 1.63) in R software, WGCNA was conducted to construct gene co-expression networks and identify modules of highly correlated genes. First, the expression data were transformed to a scale-free network with soft thresholding power to ensure that the network topology was scale-free and biologically meaningful. An adjacency matrix was constructed based on Pearson’s correlation coefficients between gene expression profiles, and a topological overlap matrix was generated to identify clusters of genes with similar expression patterns.

Modules of the co-expressed genes were identified through hierarchical clustering using the average linkage method. Each module was assigned a distinct color for visualization. The correlation between module eigengenes (the first principal component of the genes in a module) and the trait of interest (KOA or healthy status) was determined to identify modules significantly associated with the disease phenotype. Modules showing significant correlations were considered for further analysis. In the identified modules, genes were ranked based on their module membership (MM) and trait gene significance (GS). In the absence of specific criteria, MM > 0.7 and GS > 0.3 were selected as the thresholds for hub genes, indicating their central role within the module and significant association with the disease phenotype. The chromosomal locations of KOA-related EFRDEGs (KOA-EFRDEGs) were illustrated using the “circlize” (version 0.4.15) package. The online tool ImageGP (http://www.ehbio.com/ImageGP/) was used for visualizing the results of WGCNA (22).

### 2.4 Construction of PPI and transcription factor networks of KOA-EFRDEGs

PPI and TF networks were constructed and analyzed to identify key regulatory hubs and their potential roles in KOA pathogenesis. The STRING database (version 12.0) was used to construct a PPI network. The network was visualized using Cytoscape (version 3.10.2), and interactions with a combined score of >0.7 were considered significant, which ensured the reliability of the interactions. Network topology parameters, such as degree, betweenness, and closeness centrality, were calculated to identify hub genes. The “cytoHubba” plugin in Cytoscape was used to prioritize the most influential genes in the network based on their network connectivity. Potential TFs regulating the DEGs were predicted using the ChEA3 database (https://maayanlab.cloud/chea3/), a comprehensive resource for TF–target gene interactions. TFs and their target genes were integrated into the PPI network to construct a comprehensive regulatory network. This integration was performed using Cytoscape, which enabled visualization of both TF gene regulatory relationships and gene–gene interactions.

### 2.5 Diagnostic model construction and validation

#### 2.5.1 Logistic regression analysis

Univariate logistic regression analysis was initially conducted to screen for DEGs among the 12 EFRDEGs showing significant association with diagnostic outcomes (P < 0.05). These genes were subsequently subjected to receiver operating characteristic (ROC) analysis. An area under the ROC curve (AUC) value of >0.5 indicated that the tests or models possessed a certain diagnostic value. Only EFRDEGs with an AUC value of >0.75 were retained for further analysis.

#### 2.5.2 Machine learning-based gene selection

Three machine learning algorithms were used for gene selection on the basis of their robustness and effectiveness in feature selection. The least absolute shrinkage and selection operator (LASSO) regression is effective in reducing model complexity by shrinking some coefficients to zero, thus facilitating feature selection. The “glmnet” (version 4.1.7) package in R software was used for performing LASSO regression. Subsequently, we used support vector machine-recursive feature elimination (SVM-RFE), a robust classifier that can iteratively remove the least important features. The “e1071” (version 1.7.13) package in R software was utilized to perform SVM-RFE. Random forest (RF), which provides an estimate of feature importance, was used for classification and regression. The “randomForest” (version 4.7.1.1) package in R software was utilized to rank the genes according to their importance in the classification of KOA samples. Each algorithm was applied to the integrated gene expression dataset. Five-fold cross-validation was used to evaluate the performance of the models.

Genes were selected according to their significance in the model. For the LASSO regression analysis, genes with non-zero coefficients were selected. For SVM-RFE, genes that contributed to the highest accuracy in the reduced feature set were chosen. For RF, genes with a relative importance score above the threshold (set at the 75th percentile of importance scores) were considered. The genes selected by the three algorithms were intersected to obtain a consensus set of the key genes. This intersection ensured that the selected genes are robust and consistently identified as important across the different machine learning methods.

### 2.6 Construction and validation of the nomogram

The intersection of genes selected by the aforementioned methods provided a consensus list of key genes. The “rms” (version 6.4.0) package in R software was utilized to construct the nomogram. Each hub gene was assigned a regression coefficient based on its association with KOA, as determined by multivariate logistic regression analysis. These coefficients were used to calculate the risk score for each gene. The nomogram graphically represented the risk score, facilitating easy interpretation of an individual’s probability of developing KOA.

The predictive accuracy of the nomogram was validated with a separate validation dataset. The AUC value was calculated to assess the discriminative ability of the nomogram. Decision curve analysis (DCA) was performed to evaluate the clinical utility of the nomogram; this analysis provides a measure of the net benefit of using the nomogram to guide patient management decisions. The net benefit was plotted against the threshold probability for various levels of risk tolerance. The curves were generated using the “rmda” (version 1.6) package in R software. The nomogram was calibrated by comparing the predicted probabilities with the observed outcomes in the validation dataset. A calibration plot was generated, and the calibration slope was calculated to determine the extent of match between nomogram predictions and the actual observations.

### 2.7 Immune infiltration analysis

CIBERSORT, a widely used algorithm for deconvoluting complex tissue expression data into constituent cell types, was used to analyze the immune cell infiltration in the combined datasets (23). This algorithm is based on the principle of linear support vector regression and utilizes a database of gene expression signatures from pure cell populations to estimate the proportion of each cell type in the mixed tissue sample. The preprocessed gene expression matrix was uploaded to the CIBERSORTX web portal (https://cibersortx.stanford.edu/). The algorithm was configured to use the default LM22 gene signature matrix, which includes signatures for 22 distinct immune cell types. CIBERSORT estimates the relative abundance of each immune cell type in the tissue samples based on the expression of the characteristic genes of the analyzed immune cell. The output of the algorithm is a matrix of cell type abundance estimates for each sample.

The results of the CIBERSORT analysis were visualized using the “ggplot2” (3.4.4) package in R software. Violin plots were generated to display the distribution of immune cell abundance estimates across the samples, and box plots were used to compare the median abundance of each cell type between the KOA and control groups. To determine the relationships between the abundance of different immune cell types and the expression of hub genes identified in the study, Spearman’s rank correlation coefficients were calculated. A heatmap was generated to visualize these correlations, and significant correlations (P < 0.05) were highlighted.

The differences in immune cell infiltration between KOA and control samples were assessed using Wilcoxon rank-sum test, a nonparametric test suitable for comparing two independent samples. A P-value of <0.05 was considered statistically significant.

### 2.8 Animal experiments

#### 2.8.1 Surgical procedure

All animal experiments were approved by the Ethics Committee of the University of South China (No. USC2024D5026). We established the anterior cruciate ligament transection (ACLT) rat model by using the methods described in previously published studies (24). Three-week-old rats were selected for the study; the experimental group underwent ACLT surgery, and the control group received the same surgical procedure, except for ligament transection. In this experiment, isoflurane was used for rat anesthesia. The rats were placed on a dedicated anesthesia platform, and a mask was used to administer the anesthetic gas. Isoflurane was delivered through inhalation, with an induction concentration set at 5%. Once the rats lost reflexes and exhibited signs of anesthesia, the concentration was reduced to 1.5–2% for maintenance. All procedures were conducted in a temperature-controlled environment to maintain the body temperature stability of rats. Twelve weeks post-surgery, synovial tissue samples were collected from the knee joints of both groups. Six samples were collected from different rats in each group. After samples were collected, all rats were euthanized with an intraperitoneal injection of 10 mg/mL pentobarbital sodium (200 mg/kg).

#### 2.8.2 Enzyme-linked immunosorbent assay

To confirm the development of inflammation in the model group, ELISA was performed using tumor necrosis factor-alpha (TNF-α) (Cat. No. CSB-E11987r; Cusabio, Wuhan, China) and IL-1β (Cat. No. JL20884; JONLNBIO, Shanghai, China) kits in accordance with the manufacturers’ instructions for reagent preparation. Briefly, reagents were equilibrated at room temperature (18–25°C) for at least 30 min. For the assay, 100 µL of the standard or sample was added to each well, mixed gently, and incubated at 37°C for 2 h. After incubation, the liquid was discarded, and the plate was dried without washing. Next, 100 µL of biotinylated antibody solution was added, followed by 1 h incubation at 37°C. After discarding the liquid, the plate was washed three times. Next, 100 µL of enzyme-conjugated streptavidin solution was added, and the plate was incubated for 1 h at 37°C. Following five washes, 90 µL of substrate solution was added to each well and incubated in the dark for 15–30 min. Finally, 50 µL of stop solution was added to terminate the reaction, and the absorbance was measured at 450 nm within 5 min by using a microplate reader.

#### 2.8.3 Immunohistochemical analysis

The entire knee joint of ACLT and Sham rats (n = 3) was sectioned and deparaffinized. The sections were then immersed in citrate buffer (pH 6.0) for antigen retrieval. After cooling, the sections were washed to complete heat-induced antigen epitope retrieval. To block endogenous peroxidase activity, the sections were treated with 1% hydrogen peroxide at room temperature for 15 min, followed by washing with PBS. The sections were then incubated overnight at 4°C with appropriately diluted primary antibodies (MerTK, Cat. No. 27900-1-AP; PTG). After washing, the sections were incubated with secondary antibodies (Cat. No. AWI0629; Abiowell, Changsha, China) for 30 min, followed by washing with PBS. The sections were then incubated with a DAB working solution at room temperature, followed by washing with distilled water. Hematoxylin counterstaining was performed, and the sections were subsequently dehydrated and mounted. Images were captured using Slideviewer (version 2.6) and analyzed quantitatively using ImageJ software (version 2.14).

#### 2.8.4 Quantitative real-time PCR

Synovial tissues were extracted from ACLT rats and Sham rats, and qRT-PCR was performed to confirm the expression of the three key genes. We also tested the “eat me” ligand milk fat globulin protein E8 (MFG-E8) and receptor MER tyrosine kinase (MerTK), both of which are key factors of efferocytosis. Total RNA was extracted using TRIzol (Cat. No. 15596026, Thermo Fisher) by the chloroform-isopropanol method, and cDNA was transcribed and synthesized using a reverse transcription kit (Cat. No. CW2569; CWbio, Beijing, China). SYBR Green PCR Master Mix (Cat. No. CW2601, CWbio) was used for qRT-PCR experiments. Primer sequences are shown in **Supplementary Table 1**.

### 2.9 Statistical analysis

Except for the identification of DEGs, all statistical analyses were performed using R software (version 4.2.1). GraphPad Prism (version 10.2.1) was used for plotting logistic regression analysis results and ROC curves as well as for constructing correlation plots and boxplots after CIBERSORT analysis. Wilcoxon rank-sum test and Spearman’s correlation analysis were utilized for determining significant differences in gene expression and immune cell infiltration. A two-tailed P-value of <0.05 was considered statistically significant.

## 3 Results

### 3.1 Workflow

**Figure 1:**
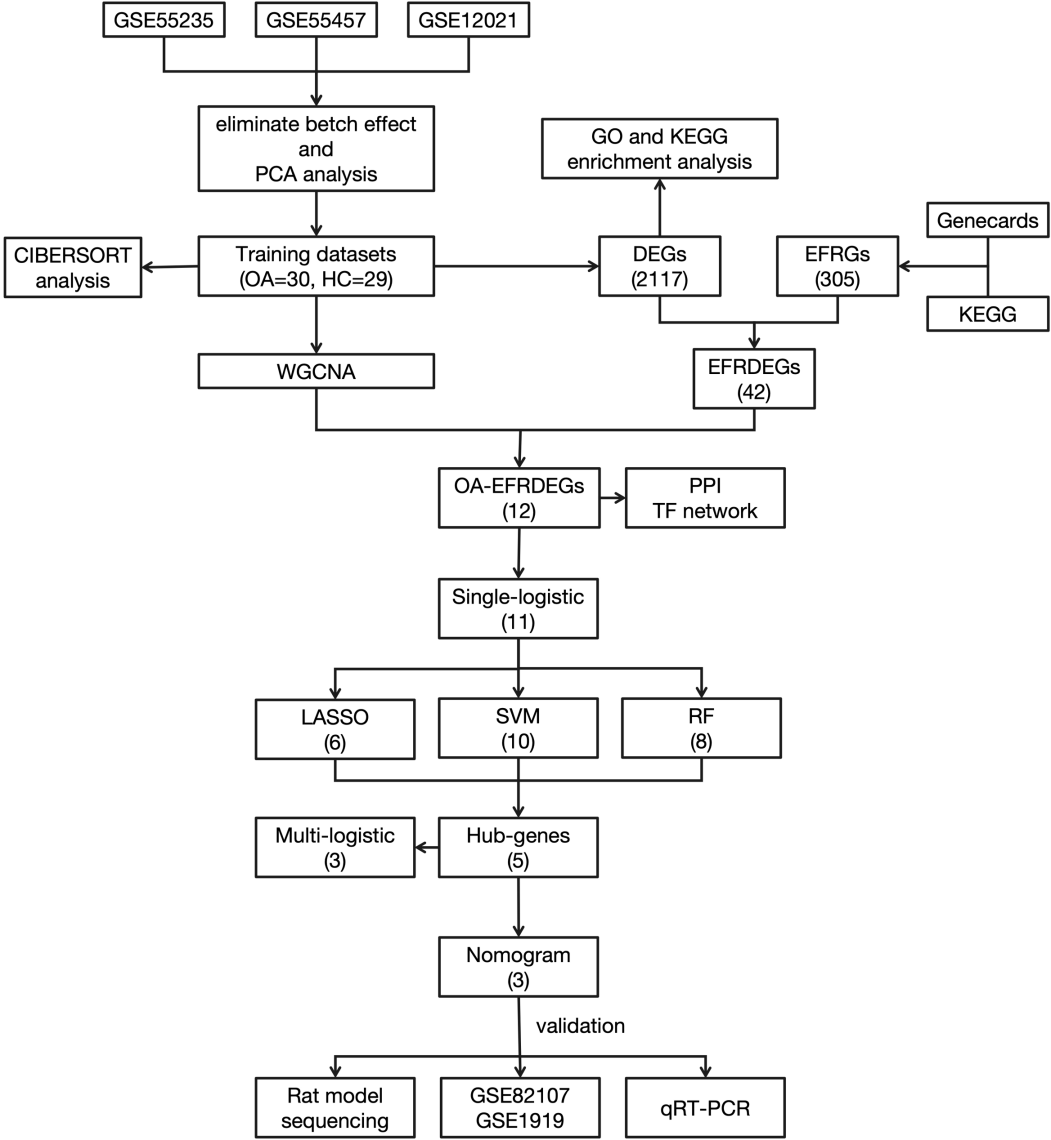
Flowchart of the study.

The workflow of this study is shown in **Figure 1**.

### 3.2 Data preprocessing, differential gene expression analysis, and GO/KEGG enrichment analysis

**Figure 2:**
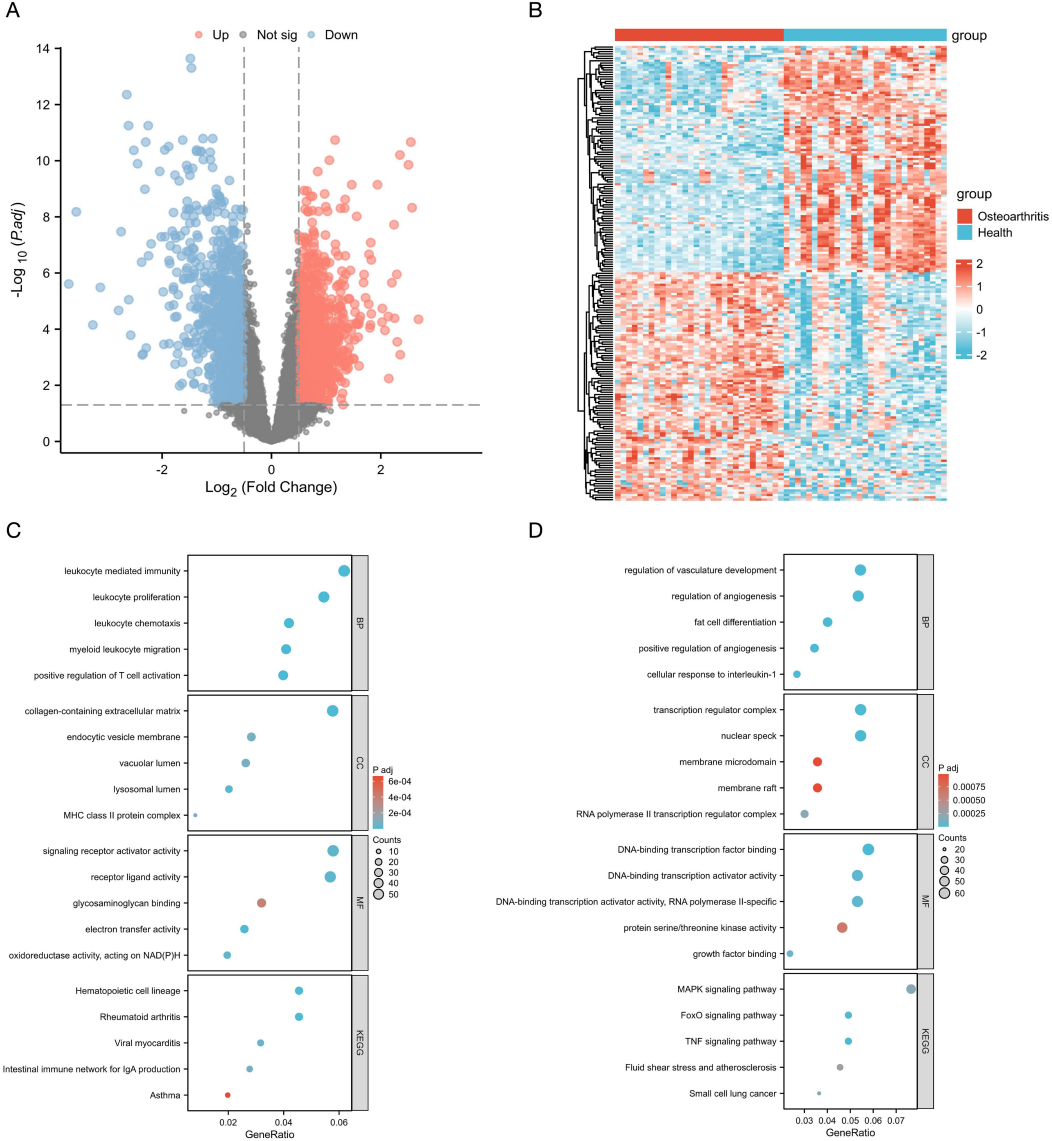
**(A)** Volcano plot of the combined training datasets. **(B)** Heatmap of the differentially expressed genes (DEGs). **(C)** The top five GO and KEGG enrichment results of the upregulated DEGs. **(D)** The top five GO and KEGG enrichment results of the downregulated DEGs.

In the training datasets, 30 and 29 samples were included in the KOA and control groups, respectively. The batch effect removal results and the PCA results before and after batch effect elimination for the three datasets are shown in **Supplementary Figure 1**. A total of 1110 DEGs were identified, with 545 upregulated and 565 downregulated genes, respectively, as shown in the volcano plot (**Figure 2A**). The clustering heatmap results are shown in **Figure 2B**.

GO enrichment analysis showed that the top 5 enriched terms of the upregulated genes in biological process (BP) were mainly involved in leukocyte-mediated immunity, leukocyte proliferation, leukocyte chemotaxis, myeloid leukocyte migration, and positive regulation of T cell activation. The top 5 enriched terms of downregulated genes in BP were mainly regulation of vasculature development, regulation of angiogenesis, fat cell differentiation, positive regulation of angiogenesis, and cellular response to interleukin-1. The abovementioned results and top 5 terms enriched in cellular components (CC) and molecular functions (MF) as well as KEGG pathway enrichment analysis results are shown in **Figures 2C** and **D**.

### 3.3 Acquisition of EFRGs

**Figure 3:**
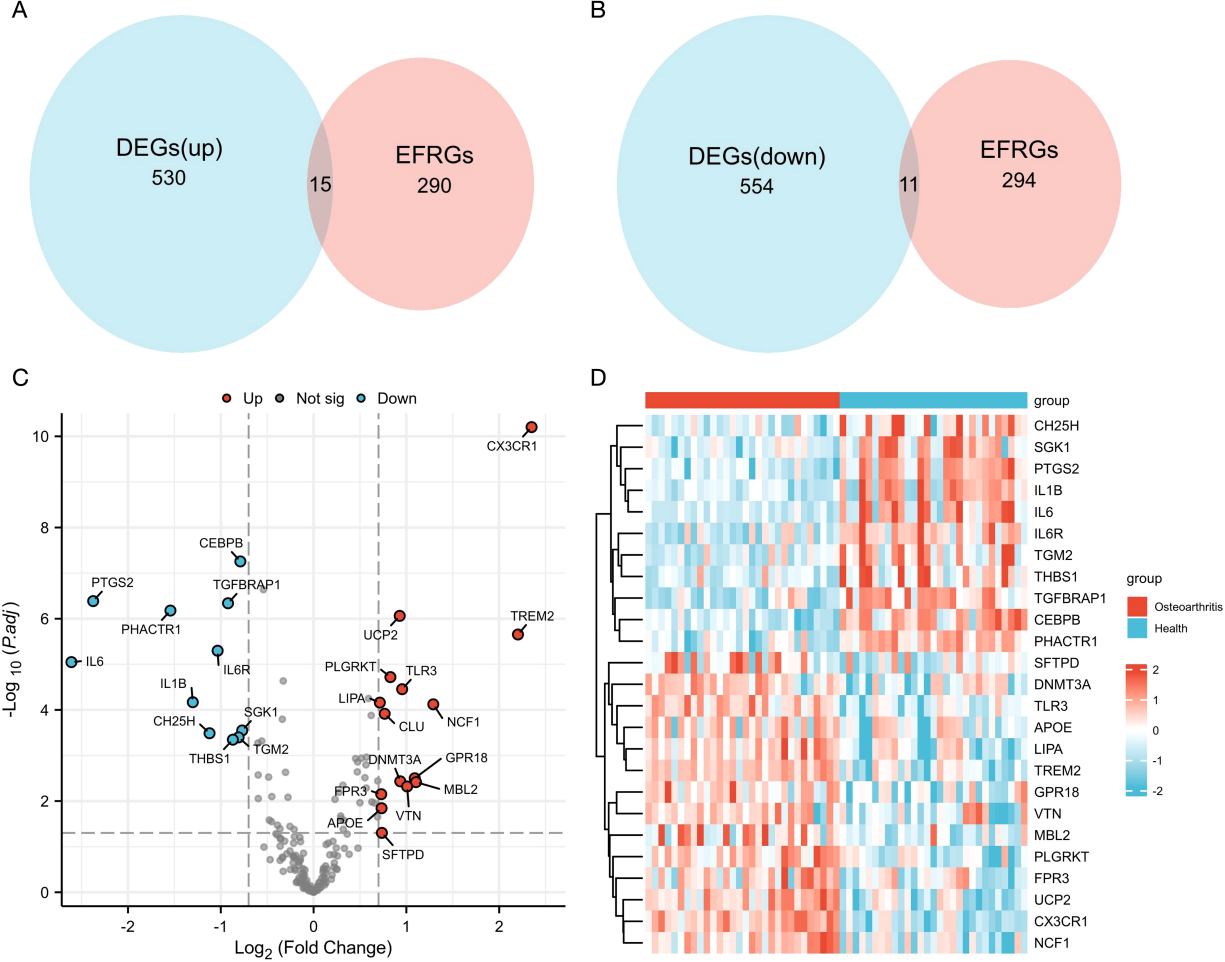
**(A, B)** Venn diagram of upregulated and downregulated genes and efferocytosis-related genes (EFRGs). **(C)** Volcano plot of efferocytosis-related DEGs (EFRDEGs). **(D)** Heatmap of EFRDEGs.

A total of 149 EFRGs were retrieved from the GeneCards database, and 131 genes were obtained from the KEGG database. Commonly utilized symbols were appended to specific molecules to ensure comprehensive inclusion of the relevant genes during the intersection process. The combination of these two lists yielded 305 EFRGs (detailed gene information is provided in **Supplementary Table 2**). The 1110 DEGs identified previously were intersected with 305 EFRGs, thereby yielding 26 EFRDEGs. Of these, 15 and 11 genes were upregulated and downregulated in the KOA group (**Figures 3A, B**). The results were visualized through a volcano plot (**Figure 3C**) and a heatmap (**Figure 3D**).

### 3.4 Weighted gene co-expression network construction and filtering of KOA-EFRDEGs

**Figure 4:**
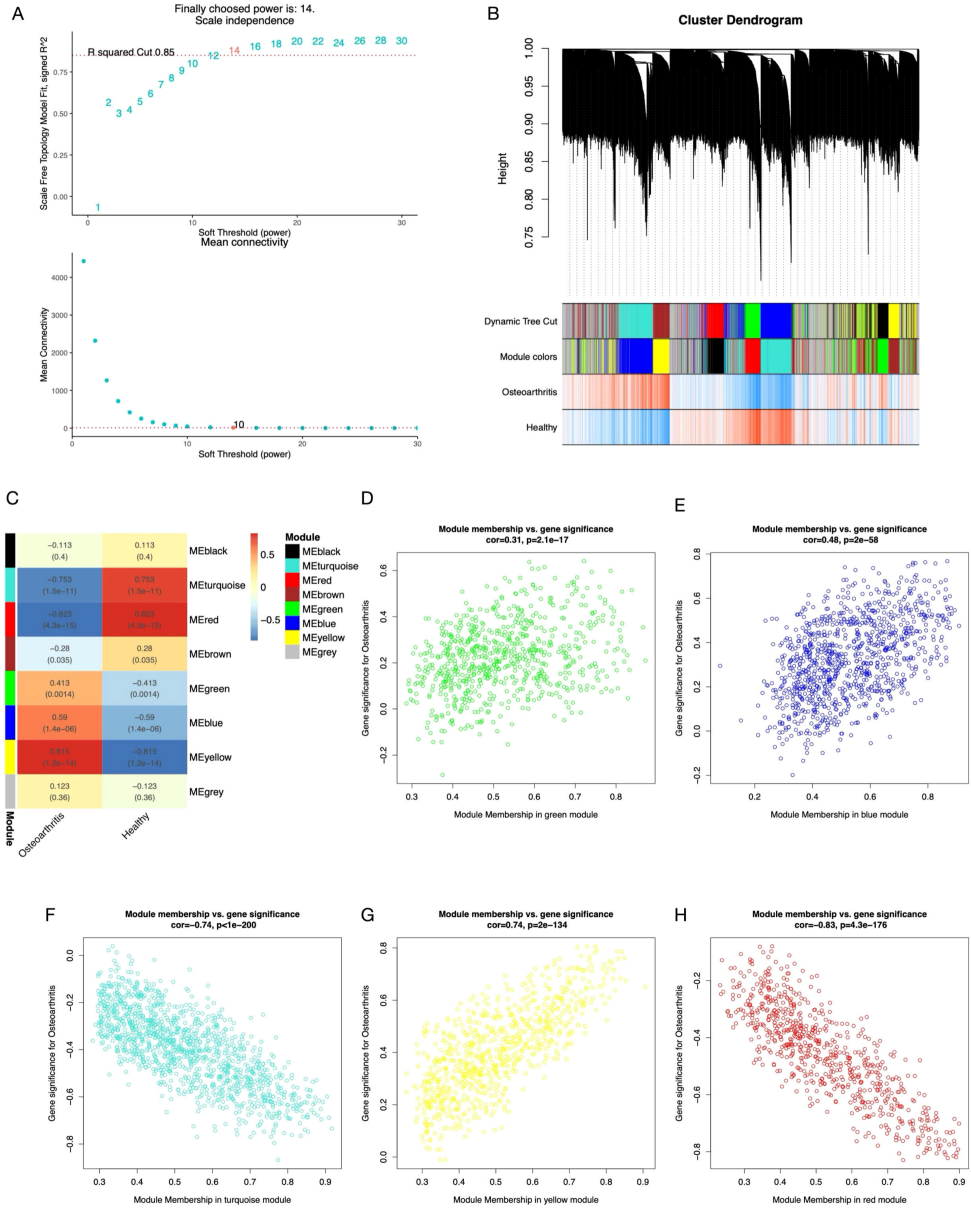
**(A)** The optimal soft thresholding power was 14 (R2 = 0.85). **(B)** Cluster dendrogram of the top 70% of genes; each branch of the graph represents a gene, and each color below represents a co-expression module. **(C)** Module–trait relationships; the minimal size of each module is 200 genes. **(D–H)** Scatterplot of correlations between gene significance (GS) and module membership (MM) in all modules with a significant correlation.

The WGCNA package in R software was used for all 59 samples in the clustering analysis. Boxplots and violin plots were utilized to visualize the abundance distribution of the samples (**Supplementary Figure 2**). To filter genes with lower variability, the top 70% of genes based on their maximum mean absolute deviation were selected; this process yielded 8862 genes for constructing the co-expression network. The optimal soft thresholding power for constructing a scale-free network was 14 (R² = 0.85) (**Figure 4A**). Clustering analysis was subsequently performed to identify and divide the highly correlated gene modules, with the minimum module size set to 200. The initial and merged modules are presented as a clustering dendrogram in **Figure 4B**. By utilizing the dynamic hybrid cutting approach, eight gene modules were obtained for further analysis (**Figure 4C**). The modules exhibiting the most significant correlation with KOA were green (716 genes), blue (994 genes), turquoise (1232 genes), yellow (770 genes), and red (688 genes) (**Figures 4D–H**). The complete module details are provided in **Supplementary Table 3**. To ensure comprehensive inclusion of key genes, those with cor.MM > 0.7 and |cor.GS| > 0.3 in these modules were designated as hub genes. These hub genes were intersected with EFRDEGs, which yielded 9 KOA-EFRDEGs for further screening.

### 3.5 PPI and TF interaction network construction

**Figure 5:**
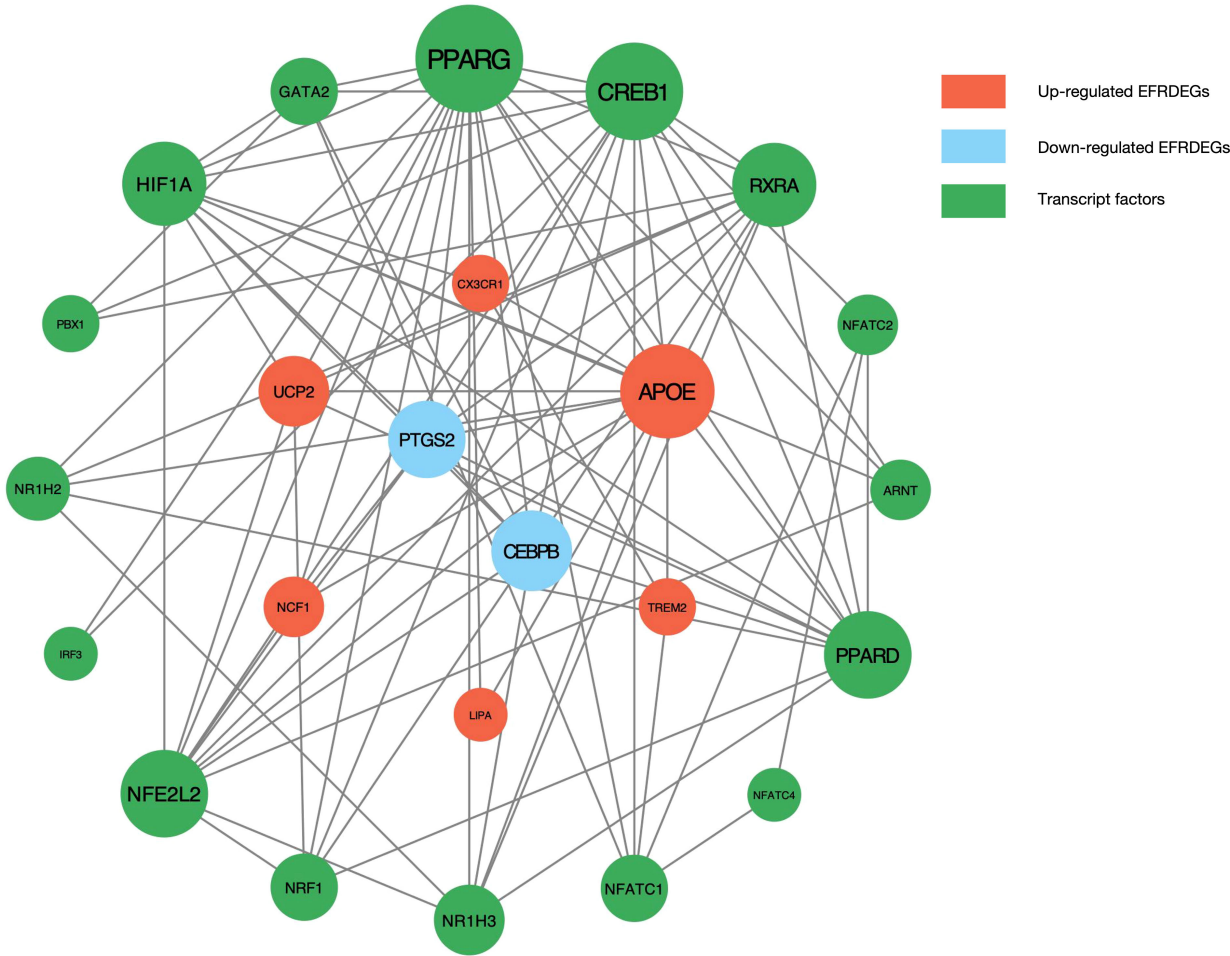
PPI network of the EFRDEGs and their possible TFs predicted by the ChEA3 database. The size of a knot depends on the number of its edges.

A PPI analysis of the 9 KOA-EFRDEGs was initially conducted with the STRING database, which excluded the PHACTR1 gene due to the absence of interactions with other nodes. Subsequently, TFs for the 8 KOA-EFRDEGs were predicted using the ChEA3 database, which yielded 1632 results. Sixteen efferocytosis-related TFs were identified through the intersection of these TFs with the EFRGs, and CEBPB, which overlapped with the key genes, was excluded. The remaining 24 TFs were analyzed using the STRING database, and the results were visualized using Cytoscape software, as illustrated in the molecular interaction network diagram (**Figure 5**).

### 3.6 Selection of diagnostic biomarkers related to efferocytosis in KOA

#### 3.6.1 Regression analysis

**Figure 6:**
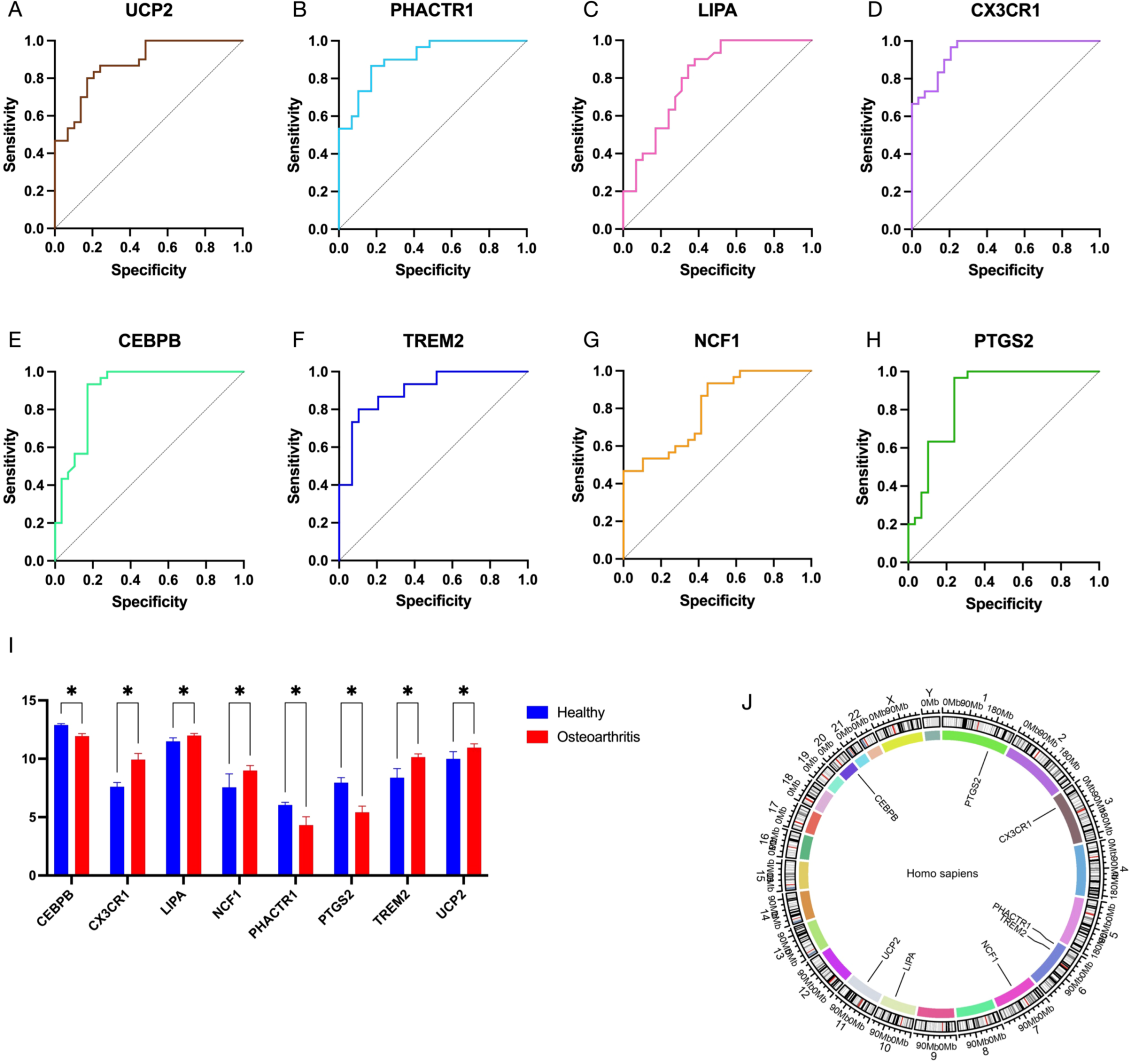
**(A–H)** Receiver operating characteristic (ROC) curves of 8 EFRDEGs selected from 12 EFRDEGs based on the criterion of AUC > 0.75. **(I)** Expression profiles of these 8 EFRDEGs in the validation dataset on the GPL570 platform. **(J)** Chromosomal location of the 8 genes. * indicates statistically significant differences between groups.

To further identify the key genes with diagnostic significance among the EFRDEGs, we initially conducted a univariate logistic regression analysis and generated ROC curves for the 9 EFRDEGs. Based on the AUC value of >0.75 as the threshold value, 8 genes, excluding APOE, were selected for the subsequent analysis. The ROC curves for these 8 genes are shown in **Figures 6A–H**, and **Figures 6I** and **J** display boxplots illustrating their expression levels and their chromosomal locations, respectively.

#### 3.6.2 Machine learning-based screening of hub KOA-EFRDEGs

**Figure 7:**
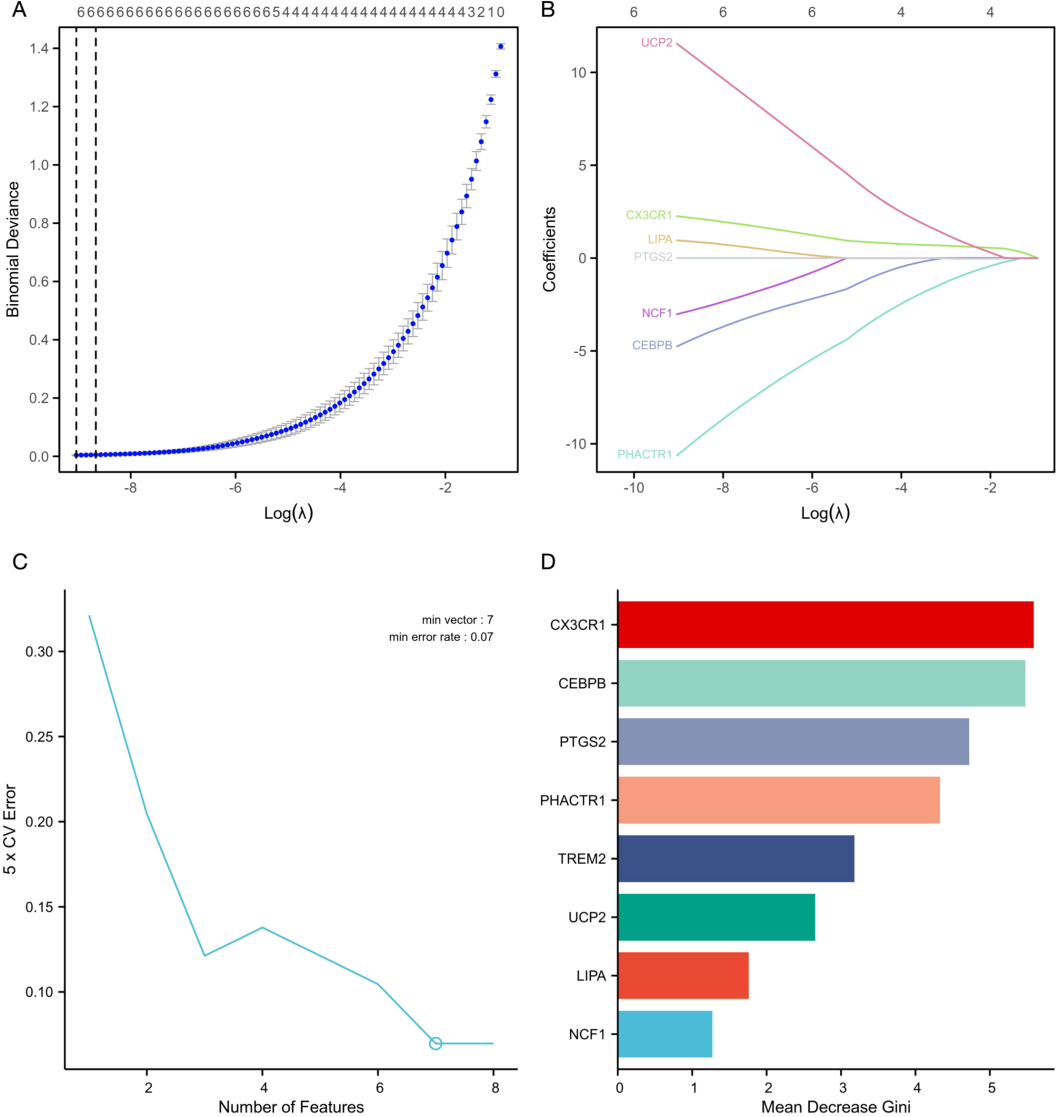
**(A)** EFRDEGs were screened using the LASSO algorithm. **(B)** The coefficient path of the key genes included in the LASSO algorithm. **(C)** SVM-RFE was used to screen the key genes. **(D)** RF plots were used to rank the importance of the included EFRDEGs.

To further screen the nine candidate hub genes identified in the previous section, we used three machine learning algorithms: LASSO regression, SVM, and RF. LASSO regression analysis with a minimum λ value of 0.00011889 identified 6 non-zero coefficient genes: *UCP2*, *CEBPB*, *LIPA*, *PHACTR1*, *CX3CR1*, and *NCF1* (**Figures 7A, B**). DCA demonstrated that SVM analysis achieved the highest accuracy (93.03%) with the inclusion of 7 genes, except NCF1 (**Figure 7C**). RF analysis identified eight key coefficients using the top 75% of mean decrease accuracy as the cutoff, which included *CX3CR1*, *CEBPB*, *PTGS2*, *PHACTR1*, *TREM2*, and *UCP2* (**Figure 7D**). By combining the results of all three algorithms, four key genes were selected: *UCP2*, *CEBPB*, *PHACTR1*, and *CX3CR1* (**Supplementary Table 4**).

### 3.7 Diagnostic prediction model construction and validation

**Figure 8:**
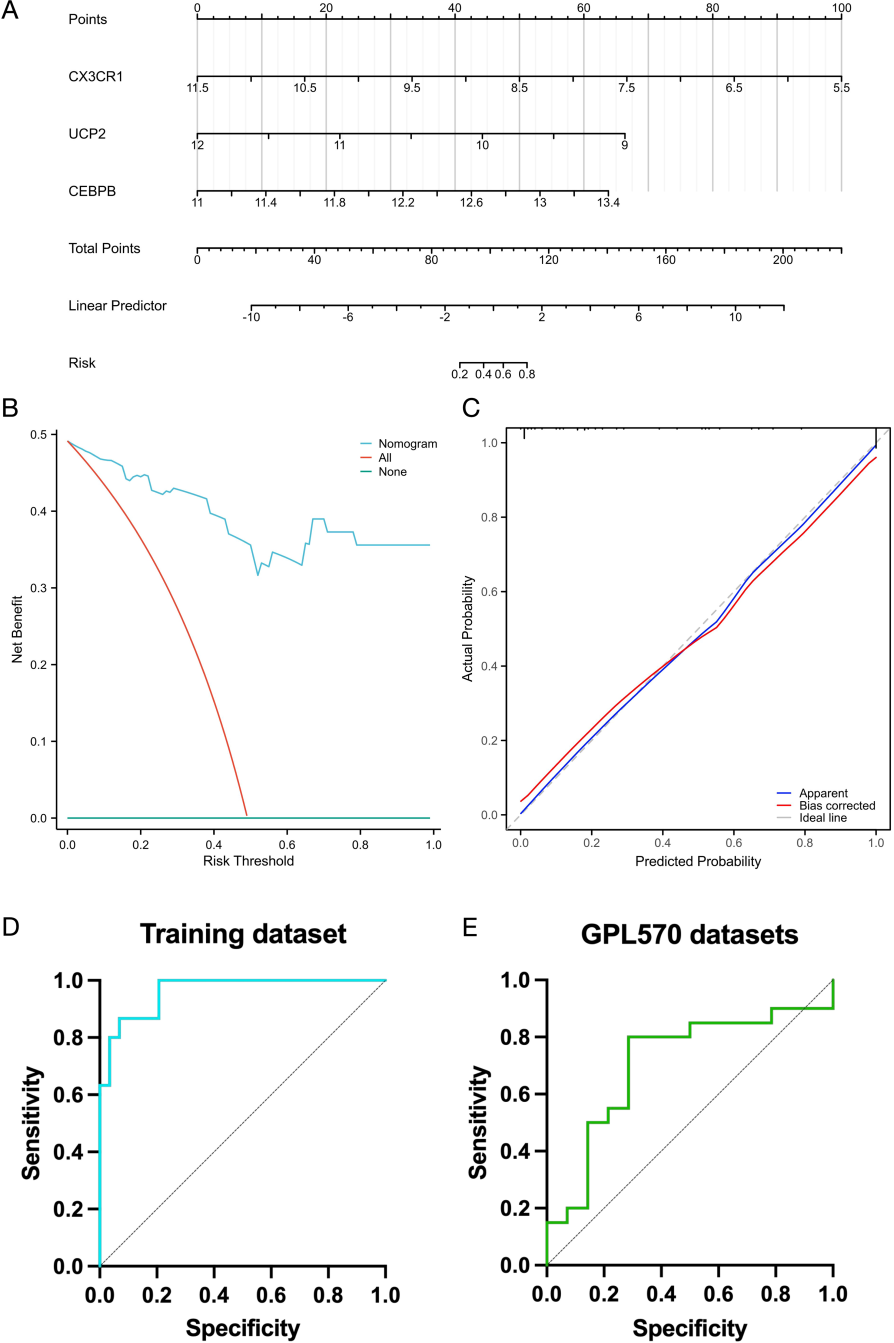
**(A)** Nomogram of 3 hub genes by using multiple logistic regression analysis. **(B)** Decision curve analysis (DCA) for the nomogram. **(C)** Calibration curves of the 3 genes. **(D)** ROC curve of the nomogram of 3 hub genes in the training set. **(E)** ROC curve of the nomogram of 3 hub genes in the validation set (GPL570 platform).

These four genes were incorporated into a multivariate logistic regression model, and *UCP2*, *CEBPB*, and *CX3CR1*, which met the threshold of P < 0.05, were finally identified as hub KOA-EFRDEGs and were included in the regression model (**Table 2**). We developed a nomogram diagnostic model for KOA based on the three hub KOA-EFRDEGs (**Figure 8A**). The nomogram integrated the expression levels of two genes, with each gene assigned a score proportional to its regression coefficient derived from the multivariate logistic regression analysis. The total score was calculated by summing the individual scores of each gene, which provided an overall risk estimate for KOA. The DCA curve (**Figure 8B**) indicated that the curves for individual genes and the overall model, derived from linear predictors, were higher than the high-risk threshold curve, thus demonstrating the high accuracy and clinical relevance of the nomogram model. The Hosmer-Lemeshow goodness-of-fit test performed with 500 resamples showed that the calibration curves in both training and test cohorts (**Figure 8C**) were close to the reference line, with a P-value of 0.98, which indicated no significant difference between the predicted and observed values and good model fit. The AUC value of the nomogram model was 0.96 (95% CI: 0.92–1.00) (**Figure 8D**), indicating high feasibility for diagnosing KOA.

**Table 2:** Related parameters of multivariate logistics regression analysis.

Characteristics	Total (N)	Univariate analysis		Multivariate analysis	
		Odds Ratio (95% CI)	P value	Odds Ratio (95% CI)	P value
CEBPB	59	76.548 (9.304 – 629.788)	< 0.001	24.929 (0.615 – 1010.140)	0.089
CX3CR1	59	0.102 (0.032 – 0.328)	< 0.001	0.133 (0.019 – 0.916)	0.040
UCP2	59	0.050 (0.011 – 0.228)	< 0.001	0.069 (0.003 – 1.797)	0.108

Subsequently, the validation cohort from the datasets of GPL570 platform yielded an AUC value of 0.71 (95% CI: 0.52–0.89) (**Figure 8E**). These findings suggest that the diagnostic model demonstrated high efficacy in differentiating between KOA patients and normal individuals.

### 3.8 CIBERSORT immune infiltration analysis results

**Figure 9:**
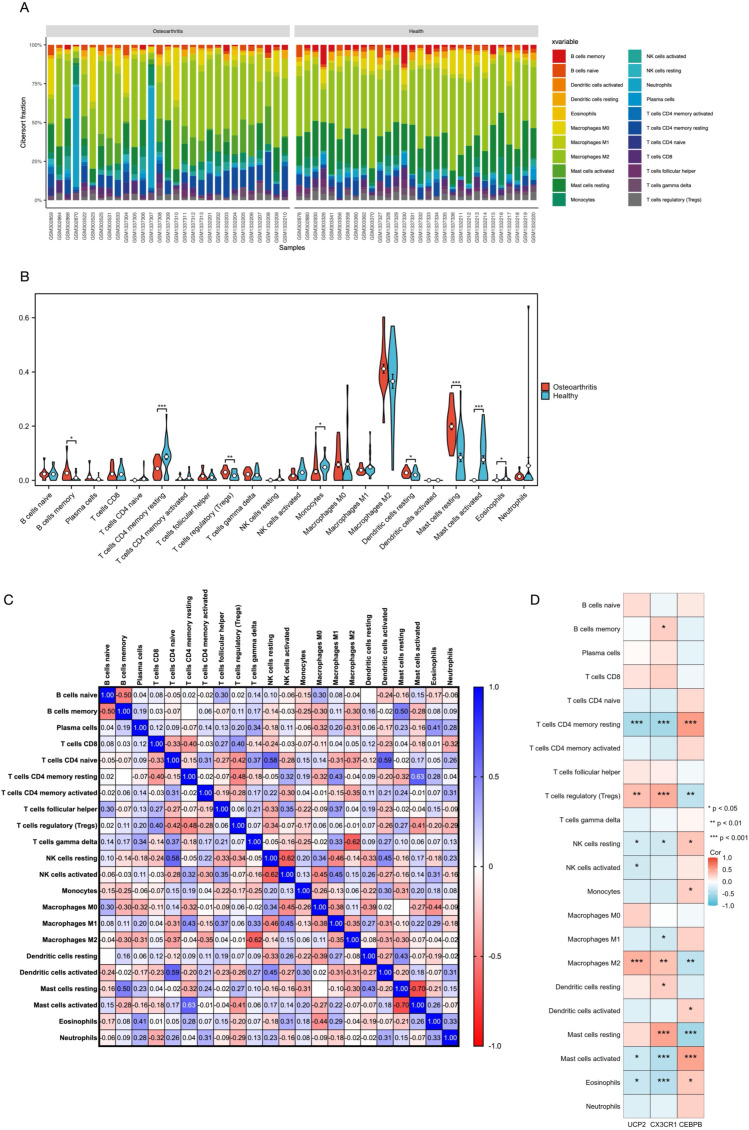
**(A)** Results of the CIBERSORT algorithm showing the proportions of 22 different immune cell types in all 59 samples. **(B)** Violin plot displaying the overall proportions of 22 immune cell types in the training dataset. **(C)** Correlation matrix illustrating the relationships between each immune cell type and all other immune cell types. **(D)** Correlations between the expression levels of 3 hub genes and the proportions of 22 immune cell types in the training dataset. * = p < 0.05, ** = p < 0.01, *** = p < 0.001.

By using 29 samples from healthy individuals and 30 samples from KOA patients in the validation cohort, the relative abundance of various immune cell subtypes was estimated using the CIBERSORT algorithm. The results are shown in **Figure 9A**. The abundance of CD4 memory resting T cells (P = 0.0005), monocytes (P = 0.0107), activated mast cells (P < 0.0001), and eosinophils (P = 0.0211) was significantly higher in KOA patients than in the healthy control group. Conversely, the abundance of memory B cells (P = 0.0047), regulatory T cells (Tregs) (P = 0.0123), resting dendritic cells (P = 0.0181), and resting mast cells (P < 0.0001) was relatively lower in KOA patients, as shown in the bar plot in **Figure 9B**. Subsequently, we determined correlations between the different immune cell types (**Figure 9C**) as well as correlations between the hub genes and various immune cell types, along with their P-values (**Figure 9D**).

The results indicated significant correlations between the three hub genes and the changes in the abundance of the eight immune cell types. Notably, *UCP2* exhibited significant correlations with seven immune cell types, with the most prominent correlations observed with T cells CD4 memory resting (r = -0.51, P < 0.0001) and macrophages M2 (r = 0.43, P = 0.0008). *CX3CR1* expression was significantly correlated with nine immune cell types, and the strongest correlation was observed with mast cells resting (r = 0.50, P < 0.0001), mast cells activated (r = -0.44, P = 0.0005), and eosinophils (r = -0.43, P < 0.0008). Similarly, *CEBPB* expression exhibited significant correlations with nine immune cell types, and the most significant correlations were found with the proportion of T cells CD4 memory resting (r = 0.54, P < 0.0001), mast cells resting (r = -0.52, P < 0.0001), and mast cells activated (r = 0.49, P < 0.0001). Furthermore, T cells CD4 memory resting, Tregs, resting NK cells, activated mast cells, and eosinophils demonstrated significant correlations with the three hub genes.

### 3.9 Resource identification initiative

**Figure 10:**
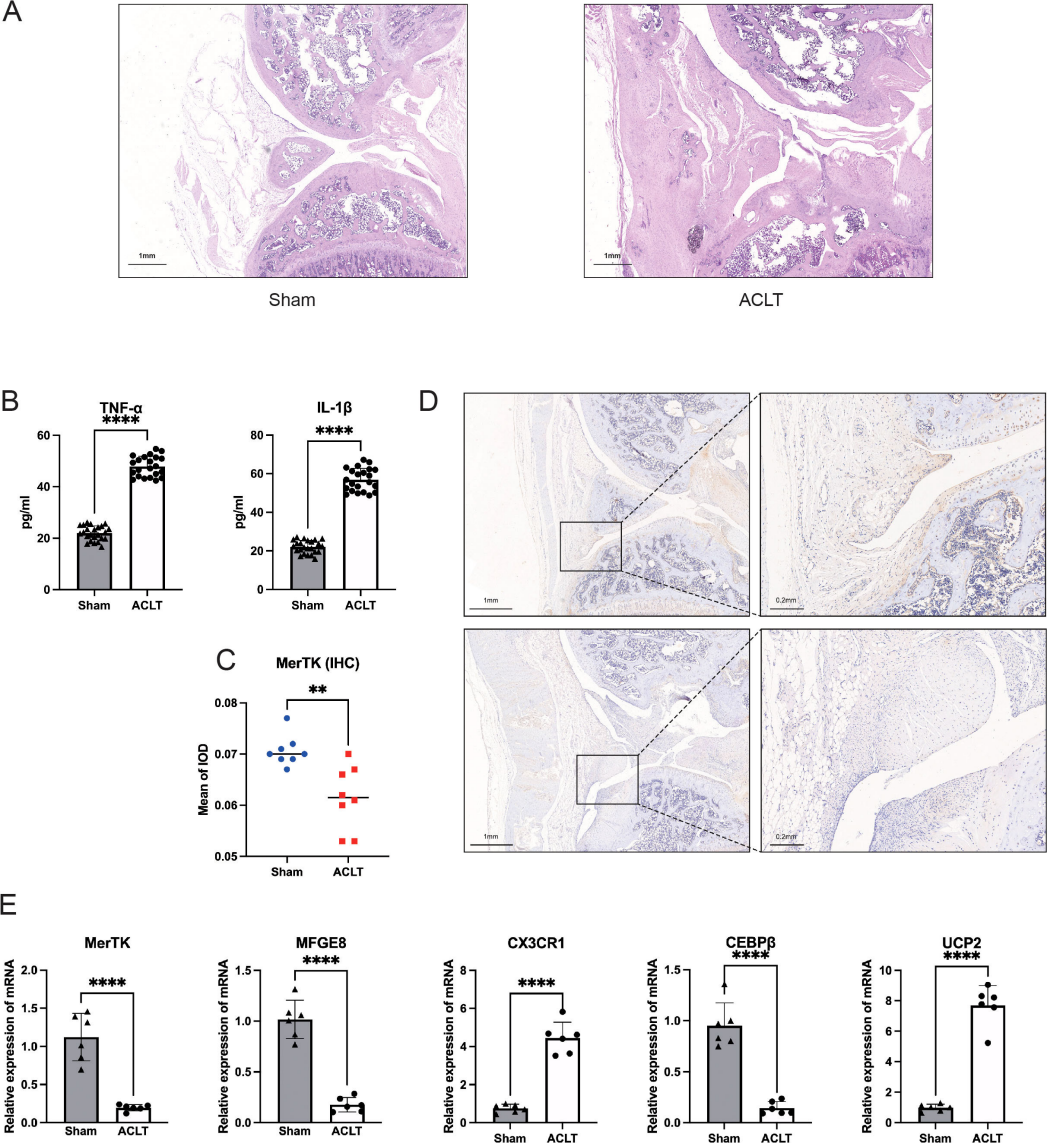
**(A)** HE staining results of joint tissue sections from the ACLT and Sham groups. Scale bar: 1 mm (5×). **(B)** ELISA results of TNF-α and IL-1β in peripheral blood from the ACLT and Sham groups. **(C, D)** IHC image of MerTK and its OD value of the two groups. Scale bar: 1 mm (5×) (left), 0.2 mm (10×) (right). **(E)** qRT-PCR results of MerTK, MFG-E8, and three hub genes. ** = p < 0.01, **** = p < 0.0001.

The results of HE staining of knee joint tissue sections from the ACLT and Sham groups confirmed the successful establishment of the KOA model. The ACLT group exhibited increased synovial tissue fibrosis, enhanced synovial tissue density, and a reduced gap between the synovium and cartilage (**Figure 10A**). ELISA results indicated that the levels of TNF-α and IL-1β in the peripheral blood of ACLT rats were significantly elevated (**Figure 10B**), which confirmed the reliability of the inflammatory model. IHC analysis and qRT-PCR results for MerTK demonstrated a reduction in its expression in the synovial tissue of ACLT rats (**Figures 10C, D**). Similarly, qRT-PCR analysis of MFG-E8 showed a comparable trend, suggesting a decline in efferocytosis levels within the synovium with the progression of arthritis. Additionally, regarding diagnostic key genes, qRT-PCR results indicated that *UCP2* and *CX3CR1* expression levels were significantly decreased in the synovial tissues of ACLT rats (P < 0.01), while *CEBPB* exhibited an opposite trend (**Figure 10E**); this finding was consistent with the expression patterns of hub genes observed in the training set.

## 4 Discussion

KOA is a classical chronic degenerative disease characterized by cartilage wear and degeneration. Traditionally, KOA has been considered a non-inflammatory condition and is often used as a control group in clinical studies on inflammatory arthritis (6). However, in recent years, the inflammatory features of KOA have received increasing attention; this is because inflammation is not merely an accompanying symptom, but it is also one of the key driving factors of the disease. Inflammation in KOA involves nearly all intra-articular tissues, with chronic, low-grade synovitis persisting throughout the disease course (25). This persistent inflammatory state has been reported to be associated with impaired efferocytosis. Synovial macrophages in KOA patients exhibit reduced efferocytotic activity, and blood-derived macrophages exposed to the synovial fluid of KOA patients display defects in this process (11). Moreover, studies utilizing local hydrogel microspheres have demonstrated that restoring *in situ* efferocytosis in cartilage tissue alleviates inflammatory progression (26). Nevertheless, the specific molecular mechanisms underlying the impairment of efferocytosis in KOA remain incompletely understood, particularly in synovial tissues.

The present study re-analyzed publicly available transcriptomic datasets of synovial tissues from KOA patients and control subjects, with an aim to comprehensively and accurately identify efferocytosis-related targets affected in KOA synovial tissue. We analyzed 3 different GEO datasets in combination with an online database to identify EFRDEGs. WGCNA and machine learning algorithms were further used to filter these EFRDEGs. Finally, we identified three key KOA predictive genes, namely *UCP2*, *CX3CR1*, and *CEBPB*, which are highly relevant to efferocytosis mechanisms, and changes in their expression levels were confirmed by qRT-PCR of ACLT rat synovial tissues. These hub genes exhibited strong diagnostic performance, with high reliability and practicality validated through public datasets. Recognizing the complexity of immune regulation involved in efferocytosis and the importance of immune response in osteoarthritis treatment (27), we conducted immune infiltration analysis by using the CIBERSORT algorithm. These findings offered novel insights for precise KOA interventions targeting efferocytosis pathways in synovial tissues, revealed compositional changes in immune cell populations within the synovial tissue of KOA patients, and highlighted the associations of these cell populations with the identified key genes.

Uncoupling Protein 2 (UCP2) is an uncoupling protein primarily involved in mitochondrial energy metabolism, with varying expression patterns across different diseases and context-dependent effects, such as inflammation. Under physiological conditions, UCP2 regulates mitochondrial membrane potential through a “mild uncoupling” mechanism, thereby reducing reactive oxygen species (ROS) production and mitigating oxidative stress (28). Tchetina et al. demonstrated a significant increase in UCP2 expression in peripheral blood, which correlated with pain in KOA patients undergoing total knee replacement. This may be due to the synergistic activation and enhancement of expression of AMP-activated protein kinase (AMPK) by an excessive amount of UCP2, leading to increased cellular energy demand. Simultaneously, the reduced affinity of UCP2 for purine nucleotides compromises energy metabolism efficiency, leading to increased pain sensitivity (29). UCP2 is also implicated in the regulation of efferocytosis through these mechanisms. By reducing mitochondrial ROS production, UCP2 controls macrophage activation. Furthermore, its role in lowering mitochondrial membrane potential enables macrophages to continuously engulf apoptotic cells without becoming overloaded (30). The present study confirmed the upregulation of UCP2 expression in the synovium of inflammatory rat models and identified the *UCP2* gene as a key parameter for diagnostic prediction. The aforementioned results provide insights into the molecular mechanisms linking this gene to KOA and efferocytosis, highlighting the potential of targeting *UCP2* as a novel therapeutic approach for KOA and related immune-mediated diseases. Future investigations should focus on evaluating the impact of gene expression modulation on disease progression and identifying the most effective therapeutic strategies. One study employed macrophage corpses as drug delivery vehicles to activate AMPK and indirectly upregulate UCP2 for the treatment of rheumatoid arthritis (31), a strategy that may also be applicable to KOA.

CX3-C motif chemokine receptor 1 (CX3CR1) is a chemokine receptor typically expressed on the surface of various immune cells, including macrophages. During efferocytosis, CX3CR1 acts as a “find me” signal. The expression of its ligand, the chemokine CX3CL1, is markedly upregulated in apoptotic cells during the degenerative phases of KOA. Through the chemokine-receptor signaling axis, CX3CL1 activates signaling pathways such as c-Raf, MEK, ERK, and NF-κB, thereby promoting inflammation and the expression of apoptotic factors such as matrix metalloproteinase (MMP)-3 (32). Following cellular apoptosis, the expression of CX3CR1 on the surface of synovial macrophages and monocytes is elevated. Simultaneously, membrane-bound CX3CL1 is cleaved into its soluble form, which induces the recruitment of CX3CR1-expressing immune cells, including mast cells, to the site of apoptotic cells (33, 34). The observed expression trends of CX3CR1 in the present study are consistent with these findings. Notably, although CX3CR1 expression increases in the OA group, it does not function as an inflammatory factor. In contrast, its upregulation is crucial for promoting efferocytosis. It cooperates with efferocytotic opsonins such as MFG-E8 and Gas6 to facilitate the recognition and contact between macrophages and apoptotic cells, further inducing phagocytosis (35). The reactive increase in the CX3CR1 level may represent a physiological anti-inflammatory feedback mechanism, supporting efferocytosis in a ligand-receptor-dependent manner to a certain extent (36). In terms of therapy, although this molecule, as a chemokine receptor, has been shown to be strongly associated with KOA, targeting it may impact multiple phenotypes simultaneously. In addition to efferocytosis and macrophage polarization, CX3CR1 also regulates chondrocyte proliferation and apoptosis (37) as well as fibroblast necroptosis (33). This complexity makes developing targeted therapies challenging, as interventions may lead to unpredictable effects on other pathways. Currently, drug research targeting CX3CR1 through various mechanisms remains at the mechanistic stage, with substantial potential for further investigation.

Interestingly, immune infiltration analysis revealed a significant negative correlation between CX3CR1 expression and the proportion of activated mast cells. Additionally, the proportion of activated mast cells was significantly lower in the KOA group than in control subjects. This may be attributed to the lower degree of synovial inflammation in KOA, where the concentration of inflammatory factors is insufficient to stimulate substantial mast cell activation. Chronic depletion of mast cells might also contribute to this phenomenon. Furthermore, a review of publicly available clinical datasets provided additional insights. In the GSE55235 and GSE55457 datasets, 16 of 26 KOA patients and 1 of 20 control subjects reported the use of nonsteroidal anti-inflammatory drugs (NSAIDs), with 20 samples included in the final analysis for each group (14). Similarly, in the GSE12021 dataset, 4 of 10 KOA patients used NSAIDs, while none of the 10 control subjects used medications (15). NSAID use suppresses the activation of immune cells, including mast cells, thereby reducing the production of inflammatory mediators, which is one of the mechanisms underlying their analgesic effects (29). However, the results of immune infiltration analysis suggest that this suppression may also partially inhibit normal immune functions.

CCAAT enhancer binding protein β (CEBPB) is a classical transcription factor involved in the regulation of immune and inflammatory response-related gene expression. It can modulate the production of multiple inflammatory cytokines, including IL-6, TNF-α, and IL-1β, and activate signaling pathways such as nuclear factor-κB (NF-κB), Janus kinase/signal transducers and activators of transcription 3 (JAK/STAT3) pathway, and the mitogen-activated protein kinase (MAPK), thereby amplifying inflammatory responses (38, 39). In KOA, CEBPB synergizes with the transcription factor RUNX2 to upregulate the expression of MMP-3 and MMP-13, thereby promoting cartilage hypertrophy and degradation (40). However, a limited number of studies have investigated the role of CEBPB in synovial tissues of KOA patients. Song et al. identified potential shared mechanisms between type 2 diabetes and KOA, which highlighted the role of *CEBPB* as a critical gene in both diseases. This aligns with our findings, as *CEBPB* expression was significantly lower in the KOA group than in the healthy control group (41). The effect of CEBPB on efferocytosis is time- and context-dependent. In late or chronic stages of inflammation, it exhibits anti-inflammatory properties by regulating the activation of macrophages (40), thereby potentially promoting efferocytosis through this mechanism. In the datasets included in this study, the average disease duration for KOA patients ranged from 6.2 to 7 years. The downregulation or depletion of CEBPB in this context may contribute to impaired efferocytosis and prolonged infiltration of inflammatory cells in synovial tissues. Immune infiltration analysis revealed a significant negative correlation between CEBPB expression and the proportion of M2 macrophages. Beyond the substantial variability caused by the dramatic shifts in the proportion of activated mast cells, this could also indicate a potential negative feedback regulatory mechanism between CEBPB expression and M2 polarization, warranting further investigation in future studies. Therapeutically, approaches targeting CEBPB function have been explored in other immune-inflammatory diseases, such as spontaneous hepatitis (42) and Alzheimer’s disease (43). These studies have validated the impact of targeting this molecule through compounds or gene editing. However, research focusing on CEBPB as a therapeutic target for KOA remains limited, representing a valuable avenue for future investigations. Subsequent studies could target its gene transcription or protein function to assess both efficacy and safety in KOA treatment.

In addition to the relationships among the aforementioned key genes, the immune infiltration analysis yielded further intriguing results. The immune cell infiltration data obtained from CIBERSORT were integrated with the findings of gene expression analysis and machine learning-based gene selection to provide a comprehensive perspective on the immune landscape in KOA. This integration enhanced our understanding of the roles played by specific immune cells in KOA pathogenesis and their potential as therapeutic targets. The results indicated that M2 macrophages, resting mast cells, and resting memory CD4 T cells had the highest proportions, which was consistent with the findings of Yuan et al. (44). Macrophages can exist in two polarization states, M1 and M2. Generally, M1 macrophages exhibit a proinflammatory phenotype during the early stages of inflammation, with their phagocytic activity primarily targeting exogenous substances such as pathogens. In contrast, M2 macrophages gradually increase during the mid-to-late stages of inflammation, secreting anti-inflammatory factors to promote tissue repair. They also play a critical role in engulfing large numbers of endogenous apoptotic cells to resolve inflammation (45). However, unlike previous studies, our results showed that the proportion of M1 macrophages was lower in the KOA group than in the control group. This discrepancy may stem from earlier studies combining datasets from cartilage and synovial tissues, whereas our results focused specifically on synovial tissue datasets. This should be confirmed by an independent analysis employing additional datasets in the future. Notably, our findings suggest an increased proportion of resting mast cells in KOA synovium, accompanied by a decrease in activated mast cells releasing histamine and other inflammatory mediators. This observation likely reflects synovium-specific immune processes, as opposed to cartilage, which exhibits acute inflammatory features in KOA (25). Additionally, medication usage among the patients providing samples may have influenced the composition of immune cells. The proportion of resting memory CD4 T cells showed a highly significant correlation with all three key genes and was reduced in KOA synovial tissue. Moradi et al. detected substantial CD4+ T cell infiltration in KOA synovial tissue; under milder inflammatory conditions, macrophages outnumbered CD4+ T cells by approximately six-fold (46), a finding consistent with our immune infiltration results. The reduced levels of activated memory CD4 T cells might still reflect the characteristics of mild inflammation. M2 macrophages also exhibited a strong correlation with the three hub genes and showed a positive relationship with inflammation severity. However, their proportion in synovial tissues did not differ significantly between the KOA and control groups. This finding suggests that, in the included KOA synovial samples, immune processes such as efferocytosis were moderately inhibited or not fully activated by the chronic inflammatory environment. Finally, it is important to note that these changes in immune cell proportions do not necessarily reflect changes in their absolute numbers. This nuance should be considered when interpreting the data and its implications for KOA pathogenesis.

The present study has certain limitations. The inclusion of patients with varying degrees of medication usage in the public datasets may have influenced the expression levels or even trends of some genes in KOA synovial tissue as well as altered immune cell composition, thereby potentially affecting the accuracy of the results. Future sequencing datasets with fewer confounding factors may help eliminate such biases. Additionally, because of the difficulty in obtaining fully suitable control synovial samples from humans, we validated the expression levels of key genes using only a rat model.

## 5 Conclusion

By conducting an integrated analysis of three public datasets, combined with EFRGs, we identified *UCP2*, *CX3CR1*, and *CEBPB* as key diagnostic genes associated with the efferocytosis phenotype in KOA. These three genes exhibited significant changes in their expression trends in knee synovial tissues, which strongly indicated the presence of KOA and abnormalities in physiological efferocytosis. Furthermore, a diagnostic nomogram was constructed, and its diagnostic efficacy was validated. The expression trends of these key genes were also confirmed through gene expression analysis in a KOA animal model. This study enhances our understanding of the molecular association between efferocytosis and KOA, guiding further therapeutic development.

## Data Availability

The authors selected the following statement: Publicly available datasets were analyzed in this study. This data can be found here: https://www.ncbi.nlm.nih.gov/geo/query/acc.cgi?acc=GSE55235; https://www.ncbi.nlm.nih.gov/geo/query/acc.cgi?acc=GSE55457; https://www.ncbi.nlm.nih.gov/geo/query/acc.cgi?acc=GSE12021; https://www.ncbi.nlm.nih.gov/geo/query/acc.cgi?acc=GSE36700; https://www.ncbi.nlm.nih.gov/geo/query/acc.cgi?acc=GSE82107; https://www.ncbi.nlm.nih.gov/geo/query/acc.cgi?acc=GSE77298.
