# Supplementary figures and images for: Identify the potential target of efferocytosis in knee osteoarthritis synovial tissue: a bioinformatics and machine learning-based study
Source: Front Immunol. 2025 Feb 27;16:1550794. doi: 10.3389/fimmu.2025.1550794 (PMC11903261; doi:10.3389/fimmu.2025.1550794)

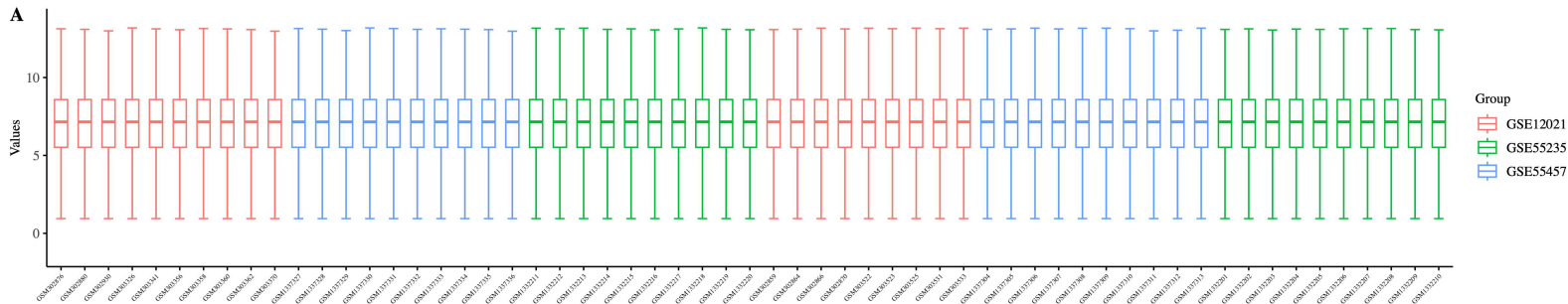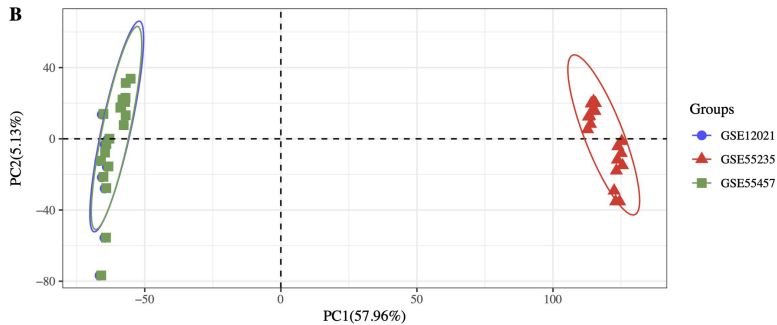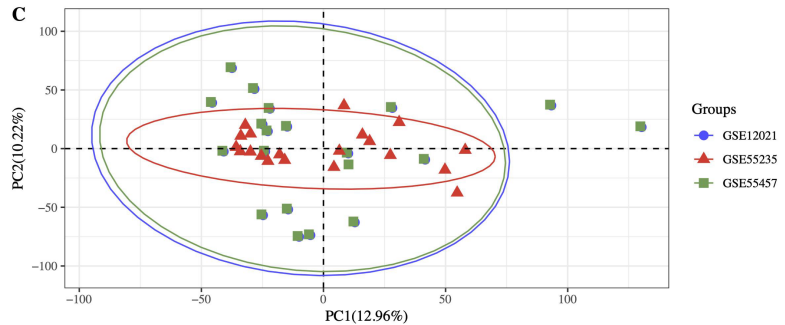

Supplement: Supplementary file 5 [file DataSheet1.pdf]

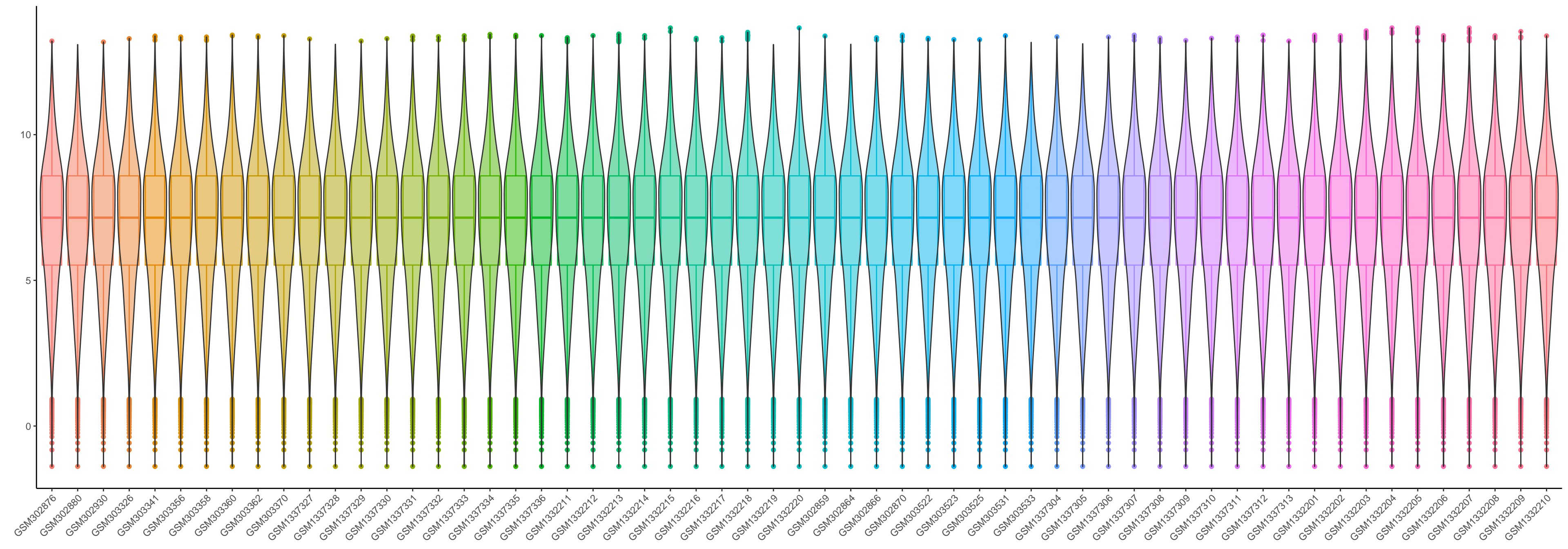

Supplement: Supplementary file 6 [file DataSheet2.pdf]
